# Long-Term Results Following Combined Repair of Atrioventricular Septal Defect with Tetralogy of Fallot

**DOI:** 10.1007/s00246-023-03343-2

**Published:** 2023-11-27

**Authors:** Katja Schumacher, Mateo Marin-Cuartas, Sabine Meier, Muhammad Ikbal Aydin, Michael A. Borger, Ingo Dähnert, Martin Kostelka, Marcel Vollroth

**Affiliations:** 1 University Department of Cardiac Surgery, Leipzig Heart Center, Strümpellstrasse 39, 04289 Leipzig, Germany; 2 Department of Pediatric Cardiology, Leipzig Heart Center, Strümpellstrasse 39, 04289 Leipzig, Germany

**Keywords:** Atrioventricular septal defect, Tetralogy of Fallot, Surgery

## Abstract

Atrioventricular septal defect (AVSD) in association with tetralogy of Fallot (TOF) is a rare and complex congenital cardiac malformation. We report our institutional experience and outcomes following surgical correction over a 20-year period. Patients who underwent combined surgical AVSD and TOF correction between October 2001 and February 2020 were included for analysis. All patients underwent primary repair. The study data were prospectively collected and retrospectively analyzed. Primary outcomes were in-hospital mortality and long-term freedom from reoperation. During the study period, a total of 10 consecutive patients underwent combined surgical AVSD and TOF correction. Median age at operation was 307 days (IQR 228–457) and median weight was 7.7 kg (IQR 6.7–9.5). Down Syndrome was present in six of the patients*.* In-hospital mortality was 0%. One patient required re-exploration due to bleeding. Median follow-up was 11 years (IQR 11 months -16 years). There was one case of reoperation due to significant residual ventricular septal defect after 2 months. None of the patients died during follow-up. Combined primary AVSD and TOF repair can be performed with low early mortality and morbidity, as well as a high long-term freedom from reoperation.

## Introduction

Atrioventricular septal defect (AVSD) in association with tetralogy of Fallot (TOF) is a rare and complex congenital malformation occurring in 5–10% of patients with AVSD [1–3]. In general, complete primary AVSD and TOF repair should be considered regardless of age. However, a two-stage approach with systemic-to-pulmonary shunt followed by delayed repair is an alternative in case of highly symptomatic neonates with high risk for cardiopulmonary bypass [1, 3–6]. Furthermore, there are still controversial opinions about the ideal repair technique, for example, regarding the number of patches used to repair the AVSD [1]. Although early postoperative mortality decreased over the last decades from 29 to 40% in 1990 to 0%–20% in recent years [2, 7], reoperation rates are still high (around 5% [8]) and remain a major issue following surgical correction [9].

The aim of this study was to analyze the early postoperative outcomes and long-term freedom from reoperation following combined AVSD and TOF repair using the two-patch technique during a time period of 20 years at Leipzig Heart Center.

## Patients and Methods

### Ethical Statement

This research project was approved by the ethics committee from the University of Leipzig. Individual patient informed consent was waived due to the anonymous data management and the retrospective nature of this study.

### Study Cohort

Patients with AVSD combined with TOF undergoing combined definitive repair between June 1999 and December 2020 were included in our analysis. Data were prospectively collected in our institutional database and retrospectively analyzed.

### Surgical Technique

Surgery was performed on cardiopulmonary bypass with aortic and bicaval cannulation in deep hypothermia. Cardioplegic arrest was achieved using St. Thomas Hospital cardioplegia solution (35 ml/kg bodyweight, repeated application of 25 ml/kg bodyweight after 90 min cardioplegic arrest).

#### AVSD Repair

AVSD repair was performed using two-patch technique in all patients. Valve exposure was obtained through a right atriotomy parallel to the right atrioventricular groove extending from the right atrial appendage to the level of the entrance of the inferior vena cava. Cold saline solution was used to identify the line of coaptation of the common atrioventricular (AV) valve and to determine the Rastelli classification.

The ventricular septal defect (VSD) was closed by sewing in a “tear shaped” Gore-Tex patch (W L Gore, Flagstaff, AZ) onto the right side of the VSD with continuous double armed 5–0 Prolene suture. Closure of the antero-superior extension of the VSD was achieved through a ventriculotomy in the right ventricular outflow tract (RVOT). The cleft was closed using horizontal interrupted Prolene 6–0 sutures. Repair of the atrial septal defect (ASD) was performed through the atriotomy with a glutaraldehyde pretreated autologous pericardial patch using running Prolene suture.

#### RVOT Repair

Through a longitudinal incision of the main pulmonary artery, the pulmonary valve was inspected and a commissurotomy was performed. Through ventriculotomy in the RVOT, hypertrophic muscle bundles were resected to enlarge the RVOT. The size of the pulmonary annulus was determined using a Hegar dilator. In case of adequate size, the RVOT was closed with a two-patch technique using autologous pericardial patch. Transannular RVOT patch was used in patients with inadequate annulus size. In 9 patients, the pulmonary valve could be preserved, and in 1 patient pulmonary valve replacement with conduit was required. After weaning from cardiopulmonary bypass, transesophageal echocardiography was performed to evaluate and document the surgical results.

### Follow-Up

Patient demographics and surgical data were obtained from the medical records from the Leipzig Heart Center. Follow-up data were collected during subsequent visits in our outpatient clinic or by contacting referring pediatric cardiologists. The degree of AV valve regurgitation was classified as mild, moderate, or severe based on the width of the color Doppler jet as evaluated in the pre-discharge echocardiography.

### Statistical Methods

Categorical variables are expressed as frequencies and percentages throughout the manuscript. Continuous variables are expressed as mean ± standard deviation for normally distributed variables and median and interquartile range (IQR) for non-normally distributed variables. Statistical analyses were performed using Microsoft Excel 2019.

## Results

### Patient Baseline Characteristics

Baseline characteristics are shown in Table 1. The median follow-up was 134 months (IQR 11–195). Among the 10 enrolled patients, all presented with complete AVSD. The Rastelli classification was determined based on echocardiographic and surgical reports [Rastelli A: *n* = 0, Rastelli B: *n* = 3 (30%), Rastelli C: *n* = 7 (70%)]. Severe AV valve regurgitation was present in 20% of the patients (*n* = 2), whereas severe RVOTO was present in 80% of the patients (*n* = 80). Median age at operation was 307 days (IQR 228–457) and median weight was 7.7 kg (IQR 6.7–9.5). Down syndrome was present in six patients (60%). Secundum atrial septal defect (*n* = 6, 60%) and patent ductus arteriosus (*n* = 4, 40%) were the most common associated congenital heart defects. A total of 4 (40%) patients underwent previous cardiac surgery prior to AVSD and TOF repair, all of them were modified Blalock–Taussig Shunt (mBTS). Table 1 depicts further information on major concomitant cardiac anomalies.

**Table 1:** Baseline characteristics

Age (days)—median (IQR)	307 (228–457)
Weight (kg)—median (IQR)	7.7 (6.7–9.5)
Female—n (%)	6 (60.0)
Previous modified Blalock–Taussig–Shunt—*n* (%)	4 (40.0)
Previous cardiac surgery—*n* (%)	4 (40.0)
Rastelli classification	
A—*n* (%)	0 (0.0)
B—*n* (%)	3 (30.0)
C—*n* (%)	7 (70.0)
Intermediate—*n* (%)	0 (0.0)
Preoperative AV valve regurgitation	
Mild—*n* (%)	3 (30.0)
Moderate—*n* (%)	5 (50.0)
Severe—*n* (%)	2 (20.0)
Preoperative RVOTO	
Moderate—*n* (%)	2 (20.0)
Severe—*n* (%)	8 (80.0)
Down syndrome—*n* (%)	6 (60.0)
Atrial septal defect type II—*n* (%)	6 (60.0)
Patent ductus arteriosus	4 (40.0)
Patent foramen ovale	1 (10.0)
Aortic anomalies	1 (10.0)

*IQR* interquartile range, *RVOTO* right ventricular outflow tract obstruction

### Intraoperative Variables

Intraoperative details are presented in Table 2. The median cardiopulmonary bypass time was 202 min (IQR 191–241), and the median aortic cross-clamp time was 135 min (IQR 118–146).

**Table 2:** Intraoperative details

CPB time (min)—median (IQR)	202 (191—241)
Aortic cross-clamp time (min)—median (IQR)	135 (118—146)
Additional procedures	
ASD type II closure—*n* (%)	7 (70.0)
PFO closure—*n* (%)	1 (10.0)
PDA ligation—*n* (%)	1 (10.0)
Temperature (celsius)—median (IQR)	24 (23—25)

*ASD* atrial septal defect, *CPB* cardiopulmonary bypass time, *min* minutes, *IQR* interquartile range, *PDA* patent ductus arteriosus, *PFO* patent foramen ovale, *TOF* Tetralogy of Fallot

### Early Postoperative Outcomes

None of the patients died within the first 30 postoperative days or during the hospital stay. Median intensive care unit (ICU) stay was 11 days (IQR 7–19). Only one (10%) patient presented with major postoperative bleeding requiring surgical re-exploration, and none of the patients required postoperative mechanical circulatory support with veno-arterial extracorporeal membrane oxygenation. No patient required a permanent pacemaker implantation. Three (30%) patients developed chylothorax postoperatively. Early reoperation was not required in any patient. In the pre-discharge echocardiography, left AV valve regurgitation was none in 1 (10%) patient, mild in 3 (30%) patients, and moderate in 6 (60%) patients. Right AV valve regurgitation was mild in 7 (70%) patients and moderate in 3 (30%) patients. Major postoperative complications are summarized in Table 3**.**

**Table 3:** Postoperative outcomes

Postoperative complications	
Chylothorax—*n* (%)	3 (30.0)
Pacemaker—*n* (%)	0 (0.0)
ECMO—*n* (%)	0 (0.0)
Bleeding requiring re-exploration—*n* (%)	1 (10.0)

Postoperative outcomes	
In-hospital mortality (%)	0.0
ICU stay (days)—median (IQR)	11 (7—19)
Hospital stay (days)—median (IQR)	20 (14—29)
Residual VSD—*n* (%)	1 (10.0)
Residual left AV valve regurgitation at discharge—*n* (%)	9 (90.0)
Mild—*n* (%)	3 (30.0)
Moderate—*n* (%)	6 (60.0)
Severe—*n* (%)	0 (0.0)
Residual right AV valve regurgitation at discharge—*n* (%)	10 (100.0)
Mild—*n* (%)	7 (70.0)
Moderate—*n* (%)	3 (30.0)
Reoperation during same hospital stay—*n* (%)	0 (0.0)
Reoperation during follow-up *n* (%)	1 (10.0)

*ECMO* extracorporeal membrane oxygenation, *ICU* intensive care unit, *IQR* interquartile range, *AV* atrioventricular, *VSD* ventricular septal defect

### Follow-Up

During the follow-up period, there was one case of reoperation due to a significant residual ventricular septal defect. Moderate left AV regurgitation remained present in 40% of the patients and right AV valve regurgitation was present in 50% of the patients. Moderate to severe pulmonary regurgitation presented in half patients.

## Discussion

The current study represents a single-center experience over a period of 20 years in patients undergoing early definitive combined AVSD and TOF repair. The main findings of our study are as follows:

1. Combined AVSD and TOF repair can be performed with very low in-hospital mortality and low rates of major postoperative complications.
2. Early and long-term reoperation rates are low.

The combination of AVSD with TOF is a rare congenital cardiac anomaly that comprises several challenges for cardiac surgeons resulting in no standardized repair approach so far.

One particularity after combined AVSD and TOF repair is the reciprocal effect of the repaired valves on each other’s function. For example, postoperative pulmonary valve regurgitation or stenosis after TOF repair is frequently diagnosed and often results in aggravated insufficiency of the right AV valve due to volume overload or right ventricular hypertension. On the other hand, left AV valve regurgitation may lead to decreased competence of the pulmonary valve with the consequence of elevated pulmonary artery pressure. The second scenario is more often detected in patients undergoing transannular RVOT patch reconstruction. These hemodynamic abnormalities increase the risk of heart failure and have therefore a relevant impact on short- and long-term outcomes [2, 10].

Furthermore, there are still discussions about the ideal age for repair surgery of combined AVSD and TOF. In these specific patients, the degree of RVOT obstruction (RVOTO) determines the time of surgery. Historical recommendations for definitive repair have been in the range of 4–6 years requiring earlier palliation in patients with severe cyanosis to allow them to reach that age [10]. In the recent decades, the recommended age decreased, and most centers perform repair surgery within the first 2 years [2, 4]. While palliation with later repair carries multiple possible complications like ventricular hypertrophy, volume overload, and progression of AV valve regurgitation, primary early repair is more likely to decrease the incidence of pulmonary hypertensive crises as well as AV valve regurgitation [2, 10]. Moreover, some surgeons argue that it is technically easier to perform the repair surgery in older patients due to more favorable anatomic features, like thicker AV valve leaflets. Furthermore, in very young or small infants, a too small sized pulmonary artery may cause difficulties to achieve an effective repair and increases the risk of main pulmonary artery re-stenosis [10]. In our cohort, we observed a change of the surgical approach over time. While the first half of the patients (operated on until 2007) initially received a mBTS prior to definitive repair, primary repair was performed in the second half of the patients (2007–2020). Additionally, patients from the second half were younger than the patients from the first half. Based on our experience, we believe that definite repair within the 1st year should be preferred over a two-stage approach, even in patients with severe RVOTO (80% in our cohort).

Another ongoing discussion is the superiority of one technique over another (i.e., one-patch versus two-patch technique). So far, there is no published comparison of these 2 techniques among patients with AVSD combined with TOF, thus no evidence supporting 1 technique over another. Since we only use the two-patch technique in our patients, we cannot make any statement about differences in outcome.

## Limitations

This study has several limitations. First, this is a single‑center retrospective study with the corresponding limitations associated with its nature. Moreover, the study does not compare different treatment strategies. Some parameters, such as the degree of regurgitation of the AV valve at discharge have been classified using qualitative echocardiographic parameters and not more precise quantitative parameters. Finally, although we exploratively looked for predictors of long-term reoperation and mortality using regression models, we were unable to identify any independent predictor, most likely due to the very limited number of patients and events of interest.

## Conclusion

Combined AVSD and TOF correction offers a safe and effective treatment alternative, with low postoperative mortality and morbidity and high long-term freedom from reoperation rates. Our current approach is to correct AVSD with TOF within the 1st year of life with no prior palliation.

## Data Availability

The data that support the findings of this study are available on request from the corresponding author.

## Abbreviations

AVSD: Atrioventricular septal defect
AV: Atrioventricular
IQR: Interquartile range
mBTS: Blalock–Taussig–Shunt
TOF: Tetralogy of fallot
RVOT: Right ventricular outflow tract
VSD: Ventricular septal defect

## References

[1] Prifti E (2017) Repair of complete atrioventricular septal defect with tetralogy of fallot. Transl Pediatr 6(1):1–7 28164023 10.21037/tp.2017.01.01 PMC5253270

[2] Hu R, Zhang H, Xu Z, Liu J, Su Z, Ding W (2014) Surgical management of complete atrioventricular septal defect with tetralogy of fallot. Ann Thorac Cardiovasc Surg 20(5):341–346 24088923 10.5761/atcs.oa.12-02198

[3] Najm HK, Van Arsdell GS, Watzka S, Hornberger L, Coles JG, Williams WG (1998) Primary repair is superior to initial palliation in children with atrioventricular septal defect and tetralogy of fallot. J Thorac Cardiovasc Surg 116(6):905–913 9832680 10.1016/S0022-5223(98)70040-6

[4] Sitaram M, Emani M, del Pedro J, Nido M (2012) Tetralogy of fallot with atrioventricular canal defect: two patch repair. Operative Tech in Thorac and Cardiovasc Surg. 17:222–235

[5] Raju V, Burkhart HM, RigelmanHedberg N, Eidem BW, Li Z, Connolly H et al (2013) Surgical strategy for atrioventricular septal defect and tetralogy of fallot or double-outlet right ventricle. Ann Thorac Surg 95(6):2079–84 23602064 10.1016/j.athoracsur.2013.02.016

[6] McElhinney DB, Reddy VM, Silverman NH, Brook MM, Hanley FL (1998) Atrioventricular septal defect with common valvar orifice and tetralogy of fallot revisited: making a case for primary repair in infancy. Cardiol Young 8(4):455–461 9855099 10.1017/s1047951100007113

[7] Prifti E, Bonacchi M, Bernabei M, Leacche M, Bartolozzi F, Murzi B et al (2004) Repair of complete atrioventricular septal defect with tetralogy of fallot: our experience and literature review. J Card Surg 19(2):175–183 15016061 10.1111/j.0886-0440.2004.04031.x

[8] Shuhaiber JH, Robinson B, Gauvreau K, Breitbart R, Mayer JE, Del Nido PJ et al (2012) Outcome after repair of atrioventricular septal defect with tetralogy of fallot. J Thorac Cardiovasc Surg 143(2):338–343 21855095 10.1016/j.jtcvs.2011.05.031

[9] Ong J, Brizard CP, d’Udekem Y, Weintraub R, Robertson T, Cheung M et al (2012) Repair of atrioventricular septal defect associated with tetralogy of fallot or double-outlet right ventricle: 30 years of experience. Ann Thorac Surg 94(1):172–178 22588011 10.1016/j.athoracsur.2012.02.070

[10] Alhawri KA, Mcmahon CJ, Alrih MM, Alzein Y, Khan AA, Mohammed SK et al (2019) Atrioventricular septal defect and tetralogy of fallot - A single tertiary center experience: a retrospective review. Ann Pediatr Cardiol 12(2):103–109 31143034 10.4103/apc.APC_87_18 PMC6521653

